# A Case of Congenital Variola in a Fœtus of Five Months, the Mother Revaccinated, Not Having Had Varioloid

**Published:** 1873-05

**Authors:** Philip Adolphus

**Affiliations:** 130 Walnut St.; Chicago


ARTICLE IV.—*A Case of Congenital Variola in a Fœtus of five Months, the Mother revaccinated, not having had Varioloid.* By PHILIP ADOLPHUS, M.D., Chicago.

Dr. Gilmore requested me to see a woman residing in Polk Street, who had spontaneously aborted a dead fœtus of five months, which, he said, was covered with small-pox pustules. This case came under my supervision as Sanitary Inspector.

Its peculiarity will at once be appreciated when the woman's history is told. Her husband had had a mild attack of varioloid some weeks previously. Dr. Gilmore revaccinated the woman above mentioned, as well as the other members of the family.

The woman remained well for ten or fifteen days, when, without having had the slightest trace of varioloid, she aborted, and a dead fœtus of five months made its appearance, covered with pustules.

I examined the woman and the fœtus.

The woman had not the slightest trace of an eruption.

The fœtus presented the following appearances: The disease was of the discrete variety; perhaps there were about a hundred pustules over the body. Having been kept from the open air, and bathed in the amniotic fluid, the pustules were whitish and flattened, and resembled the pustules met with on the mucous membrane of the mouth; many had the slight pseudo-membranous disk which covered the pustules; removed, on exposed ulcerated surfaces.

This is a case in which the fœtus only had variola before birth, though the mother had not been afflicted with it.

"Some years ago, a woman in the wards of Professor Tonquier was delivered of a dead child, affected with small-pox, although she had herself been vaccinated."

"The illustrious Mauricean relates that his mother, when in the last stage of her pregnancy, had the misfortune to lose the eldest of her three sons by small-pox, to whom, notwithstanding her condition, she was unceasing in her attentions, and that at his birth, which occurred the day after the death of his eldest brother, he presented four or five pustules of small-pox." (Cazeaux, fol. 566.)

"Ebel, Kesler, Watron, Jenner, Denent, Royer, Bouchut, and Chaigneau, have all seen children born with variola, the mothers being free of the disease. In several of these cases the mothers having been vaccinated were insusceptible to the epidemic influence, yet were able to communicate the virus to the fœtus." (Ibid, fol. 447.)

130 Walnut St.

